# Impacts of temperature and solar radiation changes in northern Europe on key population health behaviors: a scoping review of reviews

**DOI:** 10.1177/14034948231216909

**Published:** 2023-12-23

**Authors:** Heini Wennman, Timo Partonen

**Affiliations:** Equality Unit, Department of Public Health and Welfare, Finnish Institute for Health and Welfare, Finland

**Keywords:** dietary intake, diurnal preference, insomnia, seasonal, sedentary

## Abstract

**Aim:**

Climate change threatens health directly as well as indirectly through impacts on health-related behaviors. Physical activity, nutrition and sleep are key health-related behaviors for population health. We aimed at elucidating the impacts of climate change which emerge gradually on these three key health-related behaviors, particularly focusing on scenarios and projections relevant to people living in the northern Europe.

**Methods:**

We conducted a systematic literature search in three different databases in January 2023 to identify English language review articles summarizing the effects of climate change on either physical activity, nutrition, sleep, or their combination.

**Results:**

We identified 15 review articles on the topic. Data on climate change impacts on nutrition and sleep were sparse, and those on physical activity were heterogeneous. The climate in northern Europe will become warmer and sunnier in summer as well as warmer and darker in winter, which will probably increase the level of physical activity, but decrease the consumption of fruits and vegetables, as well as increase the occurrence of sleep disturbances in a population.

**Conclusions:**

**The anticipated changes in physical activity, nutrition and sleep driven by climate change influence population health and call for grass-roots action plans for adaptation.**

## Background

Climate change or global warming represents one of the greatest threats to human health and survival [1,2]. According to projection models, a global warming of +2°C compared with pre-industrial times will be reached by the year 2050. In Europe, the warming will generally be higher than the global average, with unequal distributional patterns across Europe [3]. North-western Europe will on average witness a warming less than 1.5°C, but in the Scandinavian region, a higher than 2°C warming is anticipated [3,4]. Further, in northern Europe, a relatively higher warming will take place in winter than in summer [3].

The climate in northern Europe is expected to become cloudier and wetter in the future, especially in winter [3,4]. It will result in thinner snow coverage or there will be no snow at all, where the ground would not reflect daylight to a marked extent anymore and albedo will be lost. In summer, incident solar radiation is expected to increase [4]. Solar radiation to earth’s surface is an important driver of earth’s energy balance and climate system, including the surface temperature and precipitation rates [5,6]. Solar radiation and the near-surface temperatures are positively related [7], whereas cloud coverage and solar radiation are negatively related [5].

The Intergovernmental Panel on Climate Change (IPCC) has outlined the most prominent global-warming health-related risks. For Europe, these risks include increases in mortality and illness rates because of heat and substantial losses in agricultural production [8]. Contrary to the effects of heat in southern parts of Europe, in northern Europe warmer climate favors agriculture [9]. In Europe, as elsewhere in the world, extreme heat spells and other extreme climatic events have already coincided with increased mortality [10–12]. The mortality burden in Europe due to climate change is projected to increase in the future [13], although the anticipated net effect on temperature-related mortality may remain relatively small if higher heat-related mortality will coincide with a reduction in cold-related mortality [14], but the aging population with its increasing prevalence of chronic non-communicable diseases can amplify these effects [13,15].

In addition to excess mortality, climate change directly increases the odds for morbidity and may influence health-related behaviors in a negative way [13,16]. Physical activity, nutrition and sleep are key health-related behaviors associated with health status and wellbeing in all age groups; for example, the American Heart Association listed them to be among the eight most essential components of cardiovascular health [17]. Scientific evidence shows that sedentary lifestyle, suboptimal diet and imbalanced sleep all increase not only the risk of mortality, but also the incidence rates of non-communicable diseases, especially those for respiratory and cardiovascular diseases, and the odds for cardiometabolic risk factors such as obesity and elevated blood pressure, as well as heightened blood glucose levels [18–21].

Despite evidence of the health-related benefits of proper physical activity, nutrition, and sleep, there remains room for improvement in the adherence to the recommendations for these behaviors among northern European populations. For example, the prevalence of adults meeting the physical activity guidelines is 50% in the Netherlands [22], 36% in Norway [23], and 42% of men and 39% of women in Finland [24]. The daily sleep duration among working-aged adults in Finland as well as in Norway is about 7 hours on average [25,26], whereas a widely applied recommendation is from 7 to 9 hours [27]. However, it is not unexpected that sleep problems are common, with 51% of women and 38% of men having chronic sleep problems in Norway [26] and 22% of men and 25% of women experiencing insufficient sleep in Finland [25]. Regarding the dietary patterns, examples from Denmark [28] and Germany [29] show that the dietary intake of fruit and vegetables as well as fibers or fiber-rich food among adults was insufficient, whereas that of meat exceeded recommended levels.

Recently, Chevance et al. [30] published a literature review on the impacts of climate change on health-related behaviors in which they included physical activity, nutrition, and sleep. The authors proposed a two-way interaction between climate change and health-related behaviors where climate change is affecting population health both directly and indirectly. In return, health-related behaviors might function as both mitigating and adaptive factors, as they have both positive and negative impacts on climate change counteractions. Excess meat consumption for example is a factor driving climate change [31]. Although comprehensive in many aspects, the review by Chevance et al. [30] was not systematic.

### Aim

Our aim was to review what is known and what is predicted as impacts of climate change on physical activity, nutrition, and sleep behaviors, with a particular focus on scenarios and projections relevant to people living in northern Europe. Herein, we decided a priori to focus on impacts which would emerge gradually due to incremental changes in ambient temperature and solar radiation, thus not to include impacts which would emerge from extreme weather events or the awareness of climate change. The impacts of climate change of our interest were defined as the effects of exposure to increases in temperature as well as exposure to changes in solar radiation, as these are the two key phenomena of climate change with the formulated predictions for northern Europe and high relevance to people living at these northern latitudes [4,6]. We posed our primary research question: What is known about the impacts of climate change on physical activity, nutrition, and sleep relevant to people living at northern latitudes of Europe? By systematically mapping and describing findings in the published reviews as well as identifying gaps in earlier research on climate change in relation to the three key health-related behaviors, our review informs future actions aimed at enhancing population physical activity, nutrition, and sleep under a changing climate.

## Methods

Our study followed the updated methodological guidelines for the conduct of scoping reviews [32] as well as the Preferred Reporting Items for Systematic Reviews and Meta-Analyses guidelines [33]. The review protocol was not pre-registered.

The Medline Ovid, Cochrane Libraries, and Web of Science databases were systematically searched in January 2023 for English-language review studies. One search for each of the three health-related behaviors (physical activity, nutrition, and sleep) was performed in each database. Search terms were restricted on the abstract, title, author keywords, and subject terms (MESH terms). The full search strategies are presented as supplementary material (see Supplementary notes). A reference list screening (i.e. a backward reference search) was performed on the full-text articles which met the inclusion criteria and was identified through the database searches. The articles found from the backward reference search were evaluated against the inclusion criteria as well.

### Study selection

Both authors participated in the title and abstract screening as well as in the full-text screening to identify potentially relevant articles. Studies which met all the following criteria were included: (a) aim: to summarize the effects of climate change on physical activity, nutrition, or sleep behavior in humans; (b) type: systematic review, meta-analysis, scoping review, or literature review; (c) year of publication: 2013–2023; (d) language: English; and (e) results applicable to northern Europe.

Studies were excluded if they did not report findings which described the impacts of climate change on at least one of the three health-related behaviors. Typical reasons for exclusion were: studies reporting mitigating effects of food consumption or physical activity on climate change; where studies were policy actions for stopping climate change; and studies concerning only a specific population or part of a population not applicable to northern Europe.

### Data extraction and evidence synthesis

Data on study characteristics (e.g. authors, year of publication, type of the review, aim of the review, number of original articles included in the review, and main findings) were extracted and tabulated in a Microsoft Excel (Office 365) datasheet. All results considering the impact of climate change on physical activity, nutrition, or sleep were initially identified. However, as the focus of our review was on temperature and solar radiation, we summarized only these findings in the final tables and our synthesis.

## Results

**Figure 1.**
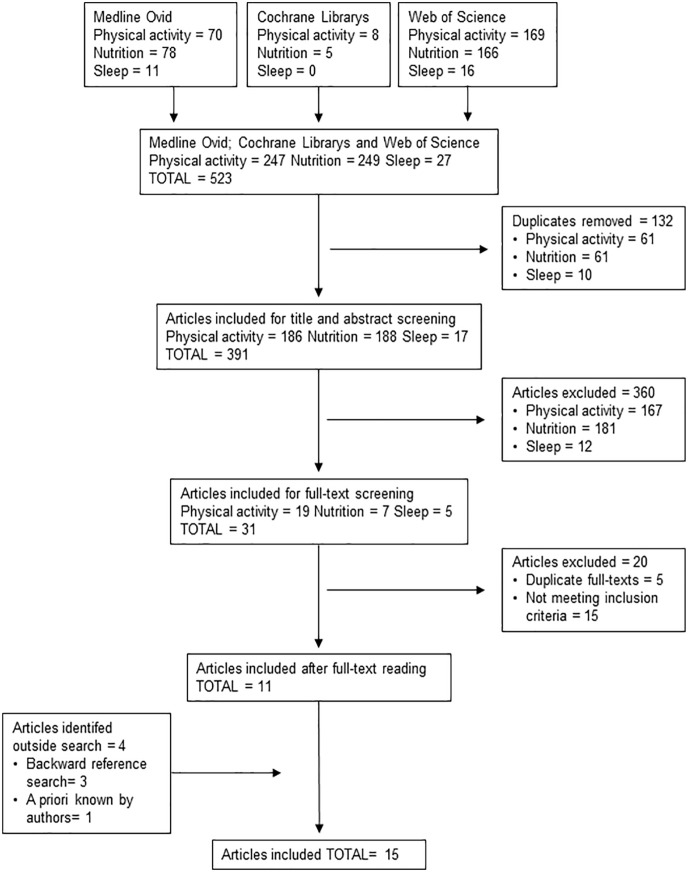
PRISMA flowchart of the study selection process. PRISMA: Preferred Reporting Items for Systematic Reviews and Meta-Analyses.

As presented in the flowchart (Figure 1), there was a total of 523 references identified in the systematic search of the three databases. After removal of duplicate references within health behavior categories and screening the titles and abstracts, 26 articles were included in the full-text assessment. Eleven articles were accepted based on the full-text assessment, in addition to which four articles were identified as relevant in the backward reference searches and by a priori knowledge of the authors. Thus, the final sample included 15 review articles of which 6 focused on physical activity, 2 on nutrition, 2 on sleep, 1 on physical activity and nutrition, 1 on nutrition and sleep, 2 on physical activity and sleep, and 1 on physical activity, nutrition, and sleep. More details about the included reviews are given in Table I.

**Table I.** Descriptive information of the included reviews (k = 15).

Reference	Type of study	Aim of the study	Comments
Physical activity An et al. [34]	Systematic review	To systematically identify and review the empirical work that projected the future influence of global warming on people’s physical activity patterns	Included only projection studies k = 10; published between 2005 and 2020; northern Europe, north America and Australia, Mexico; not any studies on young children (<11years)
Bernard et al. [35]	Systematic review	(1) Present potential bidirectional associations between climate change impacts and physical activity behaviors in humans; (2) propose a conceptual model of climate change and physical activity	Included 31 observational, 9 experimental, 25 scenario-based models, 5 conceptual, 4 systematic review studies; majority of studies from North America and the UK, also Europe, South America, Asia, and Australia; no further information about the characteristics of the included studies
Evans et al. [37]	Literature review (narrative)	Explore the projected behavioral impacts of global climate change created by elevated temperature, extreme weather, and increased air pollution	Where prior reviews exist their conclusions were summarized, focusing on more rigorous research designs, and updating them when possible In case there were no reviews, existing trends were summarized, emphasizing more rigorous research designs
Jia et al. [38]	Systematic review and meta-analysis	To review associations between rainfall, temperature, sunlight, natural disasters, flood and drought, and weight-related behaviors and childhood obesity	Included 5 cross-sectional studies and 1 longitudinal observational study; publication years 2001–2016; the USA (3), Canada (1), the Netherlands (1), Papua New Guinea (1); children up to 18 years
Turrisi et al. [40]	Scoping review	To examine associations between device-based measures of physical activity, sedentary behavior, and weather-related phenomena	Included 110 observational studies from 30 high- and middle-income countries; publication years 2008–2020; all ages of participants; examined both season and weather correlates
Wallace et al. [41]	Integrative review	To assess how increased global temperature due to climate change may alter current recommendations for physical activity participation	Included 7 experimental or quasi-experimental studies on physical activity and heat, 6 reviews or commentaries on physical activity and heat, 6 studies on climate change and increasing average global temperature; publication years 2003–2018; included much focus on physiological effects of heat
Nutrition An et al. [43]	Systematic review	To systematically review the relationship between global warming and the obesity pandemic	Included 50 articles (20 original studies, 30 commentaries); publication year ranging 2002–2017
Binns et al. [44]	Literature review (narrative)	To consider the ways in which climate change is altering food supply and how these changes will relate to dietary guidelines in the future	No explicit information about studies that results are based on (referring to IPCC reports over 15 years)
Sleep Bragazzi et al. [46]	Narrative review	To offer a way to re-read/re-think sleep medicine from a planetary health perspective	No explicit information about studies that results are based on
Rifkin et al. [47]	Systematic review	To evaluate how climate change impacts human sleep	Included 16 observational studies; publication years 1995, 2008–2017; 9/16 from the USA, 4/16 from Europe, 2/16 from Asia, and 1 from Australia
Physical activity and nutrition Cuschieri et al. [36]	Narrative review	To explore various interactions between climate change, obesity and COVID-19 and the inauspicious future that will befall our planet unless actions are taken	No information about studies that results are based on Refers to results from An et al. 2018 (nutrition)
Physical activity and sleep Koch et al. [39]	Scoping review	(1) To map research on use of off-the-shelf wearables for measuring direct health effects of and individual exposure to climate change-induced weather extremes; (2) to examine current approaches to wearable use in this field; (3) to identify gaps in the research	Included 53 studies in 56 articles (case studies, observational studies, non-randomized and randomized controlled trials); small sample sizes (median *N* = 39); many studies conducted in laboratory settings; all ages; publications years 2013–2021; mainly high- or upper-middle-income countries; more than half from North America
Zisis et al. [42]	Mini-umbrella review	(1) To summarize the findings of systematic reviews exploring the topics of climate change and 24 h movement behaviors and/or health; (2) to elaborate on the mechanisms linking climate change, 24 h movement behaviors and health	Systematic reviews k = 8; years of publication 2013–2021 Refers to results from Rifkin et al. (sleep)
Nutrition and sleep Rocque et al. [45]	Overview of systematic reviews	To map the climate impacts, health outcomes, and combinations of these that have been studied, and to synthesize key findings	Included 94 systematic reviews; publication years 2007–2019; most studies (k = 68, 72%) with a global focus; most studies (k = 69, 73%) no specific population of interest; health outcomes in studies infectious diseases (k = 41), mortality (k = 32), respiratory, neurological and cardiovascular (k = 23), healthcare systems (k = 16), mental health (k = 13), pregnancy and birth (k = 11), nutritional (k = 9), skin diseases and allergies (k = 8), occupational health and injuries (k = 6), others including sleep (k = 17). Refers to results from Rifkin et al. (sleep); An et al. 2018 (obesity)
Physical activity, nutrition, and sleep Chevance et al. [30]	Literature review (narrative)	(1) Present information on climate change and how climate change (rising temperature, extreme weather events, air pollution and rising sea level) is shaping our health-related behaviors; (2) illustrate how promotion of health behaviors could aid climate change mitigation and adaptation; (3) provide insight into how different types of equity should be addressed when thinking about the associations between climate change and health behaviors	Comprehensive although not systematic Includes studies on diet, physical activity, and sleep as outcomes. Refers in results to reviews of Rifkin et al. (sleep); Bernard et al. (physical activity); Turrisi et al. (physical activity); An et al., 2020 (physical activity)

IPCC: Intergovernmental Panel on Climate Change.

### Impact of rising temperature on physical activity

There were 10 reviews assessing the impact of higher temperatures on physical activity [30,34-42]. Three of these were systematic reviews [34,35,38], and one was a mini-umbrella review of systematic reviews [42].

Overall, the impact of higher temperatures on physical activity was mixed (Table II). Inverse U-shaped associations of rising temperatures with total physical activity [30,40] as well as with leisure-time [34,35,41] and transportation physical activity [34,35] were observed, but some found a negative relationship between high temperatures or heat and total [30,39] leisure-time and transportation physical activity [36,42]. More consistent was the finding on lower levels of occupational physical activity with high temperatures. However, this outcome was mentioned only in two reviews [30,39] of which one assessed occupational physical activity indirectly through occupational productivity [30]. The findings on physical activity of moderate-to-vigorous intensity with high temperatures yielded positive, negative, and null associations [38,40], whereas those on sedentary time suggested both increases [36] and decreases [40] with higher temperatures.

**Table II.** Summary of the findings related to the impact of higher temperature on physical activity, nutrition, and sleep outcomes.

Health behavior outcome	Number of reviews	Impact of higher temperature	Points to consider
Physical activity (PA)	10 [30,34,35,36,37,38,39,40,41,42]	Total PA: 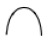 or 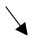 in cooler climate or winter months 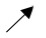 in hotter climate or summer months 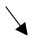 Leisure time PA: 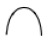 or 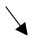 Transportation PA: 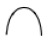 or 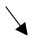 Occupational PA: 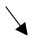 Moderate-to- vigorous PA: 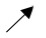 or Ø or 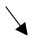 Sedentary time: 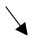 or 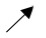	Some heterogeneity of findings between geographical locations sparse findings in children and youth Increasing temperature may increase parent’s perception of risk of their children being outdoors Those at highest risk include elderly, low socioeconomic status and those susceptible to poor health
Nutrition	5 [30,36,43,44,45]	Food supply or production: 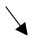 Food prices: 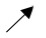 Food variety: 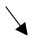 Food nutrient density or quality: 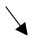	Fruit and vegetable production in particular risk Lower-income groups at higher risk of increased consumption of unhealthy foods
Sleep	6 [references: 30; 39; 42; 45; 46; 47]	Sleep duration or sleep sufficiency: 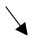 Sleep disturbances or poor sleep quality: 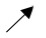 Obstructive sleep apnoea: 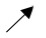	Those at highest risk include elderly, women, low-income, low socioeconomic status, poor health

Arrow upwards represents increase; arrow downwards represents decrease; Ø represents no association; inverted U stands for inverse U-shaped association.

### Findings from the systematic reviews on physical activity

Of the three systematic reviews assessing the impact of higher temperatures on physical activity, two included populations of all ages [34,35] and one included only children [38], whereas the mini-umbrella review included all age groups [42]. The two systematic reviews focusing on all age groups were similar in their conclusion, stating that rises in temperature are likely to increase the total, leisure-time and transportation physical activity levels up to a certain temperature threshold, beyond which physical activity levels will decrease [34,35]. The mini-umbrella review concluded that heatwaves will lead to decreased transportation as well as leisure-time physical activity [42]. Further, the systematic reviews concluded that the impact of temperature on physical activity may depend on the season and geographical location, so that higher temperatures in initially cooler environments will have a positive relationship with physical activity levels, whereas higher temperatures in already hot environments will have a negative relationship with physical activity levels [34,35]. The one systematic review focusing on children yielded weak and inconsistent findings [38].

### Impact of rising temperature on nutrition

There were five reviews assessing the impact of higher temperatures on nutrition and the dietary intake [30,36,43–45]. One of them was a systematic review [43], and one was an overview of systematic reviews [45]. The impact of rising temperatures on nutrition was assessed only indirectly through measures of food supply, food prices, food variety, or food quality. The findings were overall consistent (Table II), showing reductions in food supply or production [36,43–45], food variety [44], food nutrient content [30,44], or food security [30,36,45] with increasing temperatures, as well as increases in food prices [30,43].

### Findings from the systematic reviews on nutrition

The only systematic review assessing the impact of higher temperatures on nutrition concluded that a warmer climate is likely to compromise food supply and increase the prices of foods of healthier contents [43]. The overview of systematic reviews concluded that increasing temperatures will increase malnutrition globally, likely from interruptions in food production and food security [45].

### Impact of rising temperature on sleep

There were six reviews assessing the impact of higher temperatures on sleep [30,39,42,45–47]. Of them, one was a systematic review [47], one a mini-umbrella review of systematic reviews [42], and one an overview of systematic reviews [45]. All the reviews suggested that increasing temperatures will negatively influence sleep duration or sleep sufficiency, increase sleep disturbances and worsen sleep quality [30,39,42,45–47] (Table II). One review suggested that rising temperatures may increase the occurrence of obstructive sleep apnea syndrome [30].

### Findings from the systematic reviews on sleep

The one systematic review that addressed the impact of higher temperatures on sleep concluded that rising temperatures would reduce sleep duration as well as sleep quality [47]. The findings from the overview of systematic reviews [45] and the mini-umbrella review of systematic reviews [42] were both based on the results from the systematic review by Rifkin et al. [47].

### Impact of changes in solar radiation on health behaviors

No study directly assessed the impact of solar radiation on any of the three key health-related behaviors, but one review presented the effects of photoperiod on physical activity [40]. The review concluded that longer photoperiods would increase the levels of physical activity of moderate-to-vigorous intensity as well as reduce the time spent on sedentary activities.

## Discussion

With this review we mapped and described findings of 15 review articles, regarding the impacts of rising temperatures and changes in solar radiation driven by climate change on physical activity, nutrition, and sleep. Other weather-related factors such as heavy precipitation [40] may affect physical activity as well, but we decided a priori not to cover them herein. Our findings indicated that the evidence of impacts of temperature rises on nutrition as well as sleep was narrow, and heterogeneous concerning physical activity. Further, studies on the impacts of solar radiation on the three key health-related behaviors were missing from the literature, indicating a gap in knowledge and a need for research.

Here, our current scoping review not only supports, but also strengthens the conclusions presented in an earlier narrative review on the same topic [30]. We also extend those findings by including the impacts of solar radiation in the focus as well. It appears that rising temperatures will encourage and increase the levels of physical activity in a population when the temperature remains below a boundary in Celsius. However, high temperatures, and especially heatwaves, are likely to discourage and decrease levels of physical activity, so the relationship between ambient temperature and physical activity volume is not linear, but U-shaped. Further, rising temperatures will indirectly impact behavior and dietary intake because of alterations in food production, food prices, and food security. Finally, higher temperatures increase the occurrence of sleep disturbances as well as insufficient sleep in a population.

If the climate in northern Europe were to become warmer and sunnier in summertime but warmer and darker in wintertime as predicted [4], the consequences at population level on physical activity, nutrition, and sleep may include both positive and negative outcomes. An increase in physical activity due to warmer temperatures would be a positive change, since a large portion of the population fails to meet the health-enhancing physical activity levels currently. Thus far, people are physically more active in summer than in winter [40,48], but on the other hand, with warmer but darker winter days along a changing climate, people may begin to decrease rather than increase their level of physical activity [34,40,49], and since near zero (in Celsius) temperatures and precipitation make the ground slippery, people may avoid activities outdoors under such conditions [50]. The anticipated impacts pertain mainly to outdoor physical activity, which constitutes the most important source of physical activity for many, and especially older, individuals [24,51]. However, except for transportation, physical activity can be undertaken indoors as well, and it has been shown that the access to recreational facilities and locations correlates with physical activity levels, at least in adults [52]. Therefore, in case of decreasing outdoor physical activity levels due to climate change, it remains essential to ensure options open to indoor physical activity across the population.

Considering the climate change predictions together with the results of our review, it seems that the number of people who experience poor sleep is increasing, which will constitute a growing negative impact on population health in northern Europe. Ambient temperature and exposure to light are key drivers of sleep and the sleep-wakefulness cycle [53,54]. On the one hand, increases in ambient temperature directly affect sleep stages and therefore may easily disturb sleep [53]. On the other hand, warmer and sunnier summers as well as warmer and darker winters may lead to increases in misalignments of sleep schedules and therefore may easily disturb sleep, and if appropriate counter measures are not taken, higher night-time temperatures with climate warming are likely to increase the number of premature deaths from all-natural causes or non-external causes [55]. For example, in Finland, adults of the general population already tend to sleep worse in summer [56], as well as commonly have sleep disturbance to the extent of a problem in winter [57]. Sleep disorders, especially insomnia both in summer and in winter, are therefore expected to become gradually more common or more severe in a population experiencing a changing climate. Mechanistically, reduced exposure to sunlight or imbalance of exposure to light favoring the evening exposure during the day tends to delay the timing of sleep and the phase of circadian rhythms [54]. Such change in exposure to light predisposes to misalignment between the society’s timetables and the individual’s pace of circadian rhythms [25,58]. At the population level in Finland, for example, adults have already gradually delayed their diurnal preference in favor of evening hours for their daily activities during the period between the 1980s and the 2010s [25,59]. It is reasonable to expect that gradual delays in bedtime will continue, and increasingly frequent sleep disturbances will not only compromise wellbeing, but also heighten the odds for health-related hazards [58,60].

Due to climate change, the dietary patterns of people living in northern Europe may become unhealthier because of the global losses in food production and increases in food prices, but there is no evidence for direct impacts of climate change on nutrition available to support the view. Further, in addition to availability and pricing, food choices are influenced by other factors such as socioeconomic variables, together with health-related behaviors that influence nutrition [61,62]. Therefore, it remains to be explored if and how people were to change their diet and actual dietary intake following rising temperatures or changes in solar radiation due to climate change. Food insecurity is closely associated with a poorer diet quality, and this link has been amplified among low-income adults in the winter season [63]. Studies from North America and Europe show that people tend to have unhealthier dietary intakes during cold seasons, coinciding with lower levels of physical activity and subsequent weight gain [64]. Furthermore, some people in northern societies experience a marked seasonal fluctuation in appetite [57,65], with increases in appetite in winter, often emerging as carbohydrate craving and usually occurring as part of the clinical picture of seasonal affective disorder or its subsyndromal form [66]. Moreover, exposures to light or lighting conditions at the population level appear to play a role as well, since a lack of proper lighting conditions indoors was linked to a greater seasonal fluctuation in appetite and the increased odds for metabolic syndrome [67]. The burden of sleep disturbance and seasonal affective disorder as triggered by climate change will not only compromise wellbeing and increase the odds for health-related hazards, but also increase economic burden, for example, in Finland, to the magnitude of more than 5 billion euros per year [68,69].

In addition to the sparse evidence for anticipated impacts of climate change on physical activity, nutrition, and sleep, the possible scenarios are further complicated by the fact that these health-related behaviors are highly correlated. Poor sleep is known to predispose to unhealthy food choices, greater energy intakes during the evening hours, lower levels of physical activity, and more sedentary time. The longer the sedentary time, the lower the fruit and vegetable consumption is [70,71]. Furthermore, a healthy diet and good sleep link to each other [19], as do more physical activity and better sleep [72]. A behavioral change may pertain to alterations in one, in two, or all three health-related behaviors, yielding a tendency to favorable outcomes and behaviors to accumulate, and vice versa [73,74].

The most vulnerable groups to impacts of climate change on physical activity, nutrition, and sleep include the elderly, women, and those with a low socioeconomic status, as well as those with severe medical conditions [41–43,46,47]. Taking responsibility for one’s health-related behaviors requires sufficient resources; not only at an individual level but also social and societal [1]. Under a changing climate, society will face the challenge of how to guarantee resources to prevent increasingly frequent inequity in health-related behaviors [75]. Actions are needed to facilitate people changing modes of transport toward physically more active forms, their diet toward more plant-based nutrition, and their schedules to meet the need for sufficient duration and quality of sleep. Such behavioral changes might help not only in adaptation, but also mitigation of climate change [12].

Based on the observed relationships between temperature, solar radiation, and health behaviors herein, positive changes in population physical activity would also favor the mitigation of climate change. However, if population levels of transportation physical activity and outdoor physical activity do not increase, the effects on climate change are trivial. Also, increased sleep problems and unhealthier food choices due to warmer and darker climate will not slow down, but rather sustain the climate change. This further underlines the importance of all actions taken to improve population behaviors toward increasing physical activity, particularly transportation physical activity, better sleep, and a more plant-based diet, because the changing climate does not facilitate but may rather complicate these behaviors.

Our study does not come without limitations. Although the literature search was conducted systematically using guidelines, it is possible that some relevant reviews were missed, since health-related behaviors are studied in many scientific disciplines and indexed by several different terms. In addition to systematic reviews and meta-analyses we included narrative reviews that do not provide the same level of evidence as systematic reviews but may broaden the picture of what is known on the topic. Complicating the synthesis of results, the included reviews varied in their aims, where some described the relationships between weather-related phenomena and health-related behaviors [38], whereas others presented findings from projection studies [34]. We decided a priori to focus our review on two key phenomena of climate change, that is, rises in temperature and changes in solar radiation, leaving out other consequences of climate change, such as extreme weather events and increases in air pollution which occur in northern Europe as well but are more prominent in central and southern Europe [12,76]. Nevertheless, we acknowledge that population health behaviors are affected also by other factors than temperature and solar radiation, and for example, increased precipitation or heavy wind will likely decrease population physical activity [40], and extreme weather events affect food production and thereby individuals’ eating behavior [30].

## Conclusions

In conclusion, we found, first, that there were limited data on the impacts of temperature rises and changes in solar radiation on nutrition and sleep. Second, there were data but with heterogeneous findings on the impacts of temperature rises on physical activity. Due to climate change, summer months in northern Europe will become warmer and sunnier, whereas winter months will become warmer and darker, impacting the level of physical activity, nutrition by the dietary intake, and sleep. At the population level, positive impacts include increases in the level of physical activity with rising temperatures. However, higher temperature in summer and darkness and greater precipitation in winter may rule each other out. Negative impacts are forecast for nutrition and sleep at a population level, where there might be decreases in consumption of fruits and vegetables and increases in sleep disturbances and their health-related hazards.

## Supplemental Material

sj-docx-1-sjp-10.1177_14034948231216909 – Supplemental material for Impacts of temperature and solar radiation changes in northern Europe on key population health behaviors: a scoping review of reviews Supplemental material, sj-docx-1-sjp-10.1177_14034948231216909 for Impacts of temperature and solar radiation changes in northern Europe on key population health behaviors: a scoping review of reviews by Heini Wennman and Timo Partonen in Scandinavian Journal of Public Health
