# Supplementary material for: Impacts of temperature and solar radiation changes in northern Europe on key population health behaviors: a scoping review of reviews
Source: Scand J Public Health. 2023 Dec 23;53(2):184–94. doi: 10.1177/14034948231216909 (PMC11907732; doi:10.1177/14034948231216909)
Supplement: sj-docx-1-sjp-10.1177_14034948231216909 – Supplemental material for Impacts of temperature and solar radiation changes in northern Europe on key population health behaviors: a scoping review of reviews [file sj-docx-1-sjp-10.1177_14034948231216909.docx]

Supplementary notes to the Scoping Review entitled: Impacts of temperature and solar radiation changes in northern Europe on key population health behaviours: a scoping review of reviews

# Web of Science Search Strategy (v0.1)

# Database: Web of Science Core Collection

# Searches (18.1.2023)

1: TI=("climate change" OR climate OR "global warming" OR weather*) OR AB=("climate change" OR climate OR "global warming" OR weather*) OR AK=("climate change" OR climate OR "global warming" OR weather*) Results: 707958

2: TI=("physical activity" OR "physical activities" OR exercise OR sport* OR "sedentary behavior" OR "sedentary behaviors" OR "sedentary behaviour" OR "sedentary behaviours" OR "physical inactivity" OR "motor activity" OR "motor activities" OR "walking" OR "jogging" OR "running") OR AB=("physical activity" OR "physical activities" OR exercise OR sport* OR "sedentary behavior" OR "sedentary behaviors" OR "sedentary behaviour" OR "sedentary behaviours" OR "physical inactivity" OR "motor activity" OR "motor activities" OR "walking" OR "jogging" OR "running") OR AK=("physical activity" OR "physical activities" OR exercise OR sport* OR "sedentary behavior" OR "sedentary behaviors" OR "sedentary behaviour" OR "sedentary behaviours" OR "physical inactivity" OR "motor activity" OR "motor activities" OR "walking" OR "jogging" OR "running") Results: 938247

3: TI=("systematic review" OR "systematic literature review" OR meta-analysis OR "meta analysis" OR "scoping review" OR "narrative review" OR "mixed-methods review" OR "mixed methods review") OR AB=("systematic review" OR "systematic literature review" OR meta-analysis OR "meta analysis" OR "scoping review" OR "narrative review" OR "mixed-methods review" OR "mixed methods review") OR AK=("systematic review" OR "systematic literature review" OR meta-analysis OR "meta analysis" OR "scoping review" OR "narrative review" OR "mixed-methods review" OR "mixed methods review") Results: 500542

#3 AND #2 AND #1 Results: 169

4: TI=("diet" OR "dietary" OR "food consumption" OR "nutrition" OR "eating") OR AB=("diet" OR "dietary" OR "food consumption" OR "nutrition" OR "eating") OR AK=("diet" OR "dietary" OR "food consumption" OR "nutrition" OR "eating") Results: 947057

#4 AND #3 AND #1 Results: 166

5: TI=(sleep* OR "circadian rhythm" OR "circadian rhythms" OR "insomnia" OR "restless legs syndrome") OR AB=(sleep* OR "circadian rhythm" OR "circadian rhythms" OR "insomnia" OR "restless legs syndrome") OR AK=(sleep* OR "circadian rhythm" OR "circadian rhythms" OR "insomnia" OR "restless legs syndrome") Results: 306642

#5 AND #3 AND #1 Results: 16

Database: Ovid MEDLINE(R) and Epub Ahead of Print, In-Process, In-Data-Review & Other Non-Indexed Citations, Daily and Versions <1946 to January 17, 2023>

Search Strategy: 18.1.2023

--------------------------------------------------------------------------------

1 ("climate change" or "global warming" or "weather*").ti,ab,kf. (90869)

2 climate change/ or global warming/ or weather/ (39456)

3 1 or 2 (102899)

4 ("physical activity" or "physical activities" or "exercise" or "sport*" or "sedentary behavior" or "sedentary behaviors" or "motor activity" or "motor activities" or "running" or "walking" or "jogging" or "physical inactivity").ti,ab,kf. (637351)

5 exercise/ or running/ or jogging/ or walking/ or sports/ or motor activity/ or sedentary behavior/ (317814)

6 4 or 5 (750985)

7 ("systematic review" or "systematic literature review" or "meta-analysis" or "meta analysis" or "scoping review" or "narrative review" or "mixed-methods review" or "mixed methods review").ti,ab,kf,pt. (443040)

8 "meta-analysis"/ or "systematic review"/ or "Meta-analysis as topic"/ or "Systematic reviews as topic"/ (322723)

9 7 or 8 (454005)

10 3 and 6 and 9 (70)

11 ("food consumption" or "diet" or "dietary" or "nutrition" or "eating").ti,ab,kf. (800574)

12 food/ or diet/ or eating/ or "diet, food, and nutrition"/ (265231)

13 11 or 12 (902922)

14 3 and 9 and 13 (78)

15 ("sleep*" or "insomnia" or "circadian rhythm" or "circadian rhythms" or "restless legs syndrome").ti,ab,kf. (262693)

16 sleep/ or sleep deprivation/ or sleep hygiene/ or sleep latency/ or sleep stages/ or sleep wake disorders/ or sleep deprivation/ or sleep disorders, circadian rhythm/ or sleep disorders, intrinsic/ or restless legs syndrome/ or sleep apnea syndromes/ or "sleep initiation and maintenance disorders"/ (128860)

17 15 or 16 (281608)

18 3 and 9 and 17 (11)

***************************

Cochrane Libraries

Search Name: sleep

Date Run: 19/01/2023

Comment:

ID Search Hits

#1 MeSH descriptor: [Global Warming] explode all trees 0

#2 MeSH descriptor: [Climate Change] explode all trees 11

#3 MeSH descriptor: [Weather] explode all trees 6163

#4 (climate OR climate change OR global warming OR weather*):ti,ab,kw 2229

#5 #4 or #3 or #1 or #2 8215

#6 MeSH descriptor: [Sleep] this term only 4559

#7 MeSH descriptor: [Sleep Initiation and Maintenance Disorders] explode all trees 2892

#8 MeSH descriptor: [Circadian Rhythm] explode all trees 3126

#9 (sleep* or insomnia or circadian rhythm or circadian rhythms or restless legs syndrome):ti,ab,kw 58905

#10 #9 or #7 or #8 or #6 58905

#11 #10 and #5 260 /Of which 0 reviews

Search Name: nutrition

Date Run: 19/01/2023

ID Search Hits

#1 MeSH descriptor: [Global Warming] explode all trees 0

#2 MeSH descriptor: [Climate Change] explode all trees 11

#3 MeSH descriptor: [Weather] explode all trees 6163

#4 (climate OR climate change OR global warming OR weather*):ti,ab,kw 2229

#5 #4 or #3 or #1 or #2 8215

#6 MeSH descriptor: [Diet] explode all trees 20551

#7 MeSH descriptor: [Food] explode all trees 38662

#8 (diet or dietary or nutrition or food consumption or eating):ti,ab,kw 130268

#9 MeSH descriptor: [Eating] 1 tree(s) exploded 3965

#10 #8 or #6 or #7 or #9 142052

#11 #5 AND #10 740 /of which 5 reviews

Search Name: physical activity

Date Run: 19/01/2023

ID Search Hits

#1 MeSH descriptor: [Global Warming] explode all trees 0

#2 MeSH descriptor: [Climate Change] explode all trees 11

#3 #1 or #2 11

#4 MeSH descriptor: [Exercise] explode all trees 28985

#5 MeSH descriptor: [Sedentary Behavior] 1 tree(s) exploded 1358

#6 MeSH descriptor: [Motor Activity] 1 tree(s) exploded 32125

#7 (physical activity or physcial activities or exercise or sport* or sedentary behavior or sedentary behaviors or sedentary behaviour or sedentary behaviours):ti,ab,kw or #4 or #5 or #6 154911

#8 MeSH descriptor: [Weather] explode all trees 6163

#9 (climate OR climate change OR global warming OR weather*):ti,ab,kw 2229

#10 #9 or #8 or #1 or #2 8215

#11 #10 and #7 1409/ Of which 8 reviews
